# New evidence on the association of occupation with amyotrophic lateral sclerosis: A register-based case-control study in Finland

**DOI:** 10.3389/fneur.2022.859824

**Published:** 2022-09-14

**Authors:** Peppiina Saastamoinen, Hannu Laaksovirta, Päivi Leino-Arjas, Ossi Rahkonen

**Affiliations:** ^1^Department of Public Health, University of Helsinki, Helsinki, Finland; ^2^Finnish Medical Association, Helsinki, Finland; ^3^Neurocenter, Helsinki University Hospital, Helsinki, Finland; ^4^Finnish Institute of Occupational Health, Helsinki, Finland

**Keywords:** occupation, amyotrophic lateral sclerosis (ALS), case-control, epidemiology, register study

## Abstract

**Objectives:**

Amyotrophic lateral sclerosis (ALS) is a serious neurodegenerative disease that usually leads to death within a few years from diagnosis. The risk factors for ALS are still largely unknown. However, it is assumed that environmental factors play a role in disease onset. Occupation is suggested as a potential risk factor, but findings are inconsistent. The aim of this study was to assess the association of occupation with ALS in Finland. Register data were used to avoid recall bias and to obtain a large enough sample to detect the potential associations.

**Methods:**

This case-control study included ALS cases that occurred between 1980 and 2015 in Finland (*n* = 4,781). ALS cases were identified from the causes of death register. For each ALS case, six controls were selected matched for sex and birth-year. The date of death of the ALS case was set as index date. Information on occupation was obtained from Statistics Finland for all subjects. The focus was on the longest-held occupation on 2-digit level (70 groups). The association of occupation with ALS was analyzed using conditional logistic regression.

**Results:**

Compared to “clerical work and other office work,” the risk of ALS was increased in “packing and wrapping work” (OR 1.53, 95% CI 1.08–2.17), “laundering, dry cleaning and pressing work” (OR 1.83, 95% CI 1.08–3.08), and “travel service work” (OR 8.75, CI 2.76–27.74). A decreased risk was found in “planning, administrative and research work in the technical fields” (OR 0.69, 95% CI 0.48–0.98). Of the significant associations identified, only “travel service work” was significant after FDR multiple testing correction.

**Conclusions:**

This study identified occupations in which the risk of ALS was increased. Further studies are needed to pinpoint the potential exposures in these occupations that may trigger the disease.

## Introduction

Amyotrophic lateral sclerosis (ALS) is a severe degenerative neurological disease. The incidence of ALS has been ~2–3 cases/100,000 people, but a recent review suggests that the incidence is increasing in different parts of the world (1, 2). There is no effective cure for ALS, and life expectancy after the diagnosis is typically 2–5 years (3, 4).

The etiology of ALS is still largely unknown. However, the onset and progression of the disease likely occur in an interplay between genes and environment (5–9). Thus, the degenerative changes related to aging and the cumulative effect of environmental risk factors may trigger the disease.

Work–life is a potential source of exposure that could predispose to ALS. According to our knowledge, only a few large-scale register-based studies have focused on the association of occupation with ALS. Recently in Denmark, a register-based case–control study found an increased risk of ALS among men in agriculture, hunting, forestry, fishing, and construction work (10). In a Swedish register-based case–control study, a higher risk of ALS was associated with precision-tool manufacturing and glass, tile, and pottery work, while a lower risk was found in textile work (11). In a smaller-scale case–control study in the USA, an increased risk was found between construction workers and precision metal workers (12). In Italy, a follow-up study including 208 ALS patients found an increased risk among bank tellers, general practitioners, and sales representatives (13).

There is evidence that the exposure to chemicals (e.g., pesticides, formaldehyde, dry-cleaning agents), heavy metals (e.g., lead and mercury), extremely low-frequency electromagnetic fields, particulates, and combustion products and diesel exhaust is associated with an increased risk of ALS (11, 12, 14–21). The most convincing results have been found concerning exposure to lead (22–25).

As environmental and occupational risk factors are assumed to affect the onset of the disease, identification of potentially important and modifiable risk factors is important to be able to prevent the disease and to understand its etiology. To identify potential exposures related to occupations, high-risk occupations need to be identified first. The existing results concerning occupations are still inconsistent, and more research is needed to clarify the association of occupations with ALS.

In Finland, register information that covers all ALS cases, and their occupational information (over the selected period), is available for research purposes. The aim of this study is to examine whether there are occupations that are associated with an increased risk of ALS among the Finnish population.

## Materials and methods

This is a register-based case–control study. Information on ALS cases was obtained from the causes of death records of Statistics Finland for the period of 1973 to 2015. The following ICD codes were used to identify ALS cases: *ICD-10:* G12.0-G12.9, G95.8; G95.9, *ICD-9:* 3350A-B, 3351A-X, 3352A-X, 3358X, 3359, 3359X; *ICD-8:* 348.0–348.99, 349.98–349.99. Altogether 6227 ALS cases were identified during the selected period. For each ALS case, six randomly selected birth-year (date of birth ± 6 months) and sex-matched controls were obtained from the Finnish Population Register Center's Population Information system. The index date for cases and controls was set as the date of death of the associated ALS case. As the *ICD-10* code for ALS includes all motor neuron diseases, the registered data may include some non-ALS cases. To avoid bias caused by false positives, all the original death certificates, where ALS was coded as the main cause of death, were reviewed by a senior neurologist (HL) and non-ALS cases were hand-picked off the data. Typical non-ALS cases were, e.g., spinal muscular atrophy types 1–3. The total number of excluded cases was 204. From 2004 onwards, we were also able to review those death certificates where ALS was recorded as a contributing cause of death.

The original data cover all ALS cases occurring in the death registers in Finland from 1973 to 2015, but in this study, the data were restricted to those who were born between 1915 and 1963, had died in 1979 or later, and were aged 35–85 at the time of death (*n* = 4,781). The controls of excluded ALS cases were also removed from the data. The restrictions were made to select those that were within the age range to have an occupation during 1975–1993. In addition, we assumed that if the person had died because of ALS before age 35, the disease was likely not related to occupational exposure due to a potentially short exposure period.

Occupational information was received from the Statistics Finland database and was combined with the case–control data. It covers years 1975, 1980, 1985, 1990, 1993, 2000, and 2004–2015. The occupational information in 1975, 1980, 1985, 1990, and 1993 had the best coverage of occupations among both cases and controls and is used in this study. We used two-digit information on occupation resulting in 70 occupational groups. As people may have information on occupation at some or all time points (depending on e.g., age, work ability, and unemployment), we decided to select the occupation with the highest frequency during 1975–1993 in our analyses. If the person had different occupations at all time points, then we selected the centermost of the available occupations which occurred over the selected period. The exact length of the exposure was not available in the Statistics Finland registers. For controls, the observation period (occupations) ended up to the year of death of their matched ALS case (index date). Occupations first held 5 years prior to the index date were excluded from the analyses to avoid reverse causality.

### Statistical methods

Conditional logistic regression analysis, which takes into account the matching for birth year and sex by grouping observations into their matched groups, was used to study the association of ALS with occupation [odds ratios (ORs) and 95% confidence intervals (CIs)]. “Clerical work and other office work” was used as the reference group in the analyses as it unlikely includes significant physical or chemical exposures. Due to multiple comparisons, Benjamini–Hochberg procedure at 0.25 was used to estimate false discovery rate (FDR).

The main analyses were done for the whole data. Additional analyses were stratified by sex to detect sex differences (as we are aware that men and women occupy partly different occupations) and by birth cohort (those born in 1915–1934 and 1935–1963) to detect potential changes over time.

## Results

Considerably more women (51.9%) than men (48.1 %) died from ALS (Table 1). Proportionally more men than women died among those under 65 years, but among those aged 65+ the sex ratio reversed (in accordance with the underlying population; data not shown). Approximately 80% of the ALS cases had died before the age of 75. Occupation was not specified, or occupational information was not available for 12.5% of ALS cases and 12.6% of controls.

**Table 1:** Characteristics of ALS cases and controls.

	**ALS cases %**	**ALS cases *n***	**Controls %**	**Controls *n***
**Sex**				
Men	48.1%	2,300	48.1%	13,799
Women	51.9%	2,481	51.9%	14,885
**Year of birth**				
1915–1924	21.4%	1,023	21.4%	6,136
1925–1934	30.1%	1,437	30.1%	8,622
1935–1944	24.9%	1,192	24.9%	7,152
1945–1954	19.0%	906	19.0%	5,436
1955–1963	4.7%	223	4.7%	1,338
**Year of death**				
Alive 31.12.2015	0	0	62.5%	17,932
1980–1985	7.4%	352	0.3%	91
1986–1995	19.2%	918	4.0%	1,144
1996–2005	33.6%	1 606	12.0%	3,446
2006–2015	39.8%	1 905	21.2%	6,071
Age^*^				
35–44	1.7%	82	1.7%	490
45–54	10.6%	505	10.6%	3,045
55–64	29.1%	1,392	29.1%	8,346
65–74	37.6%	1,798	37.5%	10,769
75–84	20.1%	963	20.2%	5,788
85–94	0.9%	41	0.9%	246
Information on occupation available^*^/^**^				
no information or				
occupation not specified	12.5%	598	12.6%	3,613
1 timepoint	11.5%	548	11.4%	3,260
2 timepoints	16.9%	808	16.8%	4,815
3 timepoints	16.5%	790	16.4%	4,696
4 timepoints	16.6%	794	16.4%	4,711
5 timepoints	26.0%	1,243	26.5%	7,589
**Total**	14.3	4,781	85.7	28,684

* At the index date.

** Timepoints are years 1975, 1980, 1985, 1990, and 1993.

An increased risk was found for packing and wrapping work (OR 1.53, 95% CI 1.08–2.17), laundering, dry cleaning and pressing work (OR 1.83, 95% CI 1.08–3.08), and travel service work (OR 8.75, 95% CI 2.76–27.74) compared to clerical work and other office work (Table 2). In addition, for example, dock and warehouse work, mining and quarrying work, woodwork, and forestry work showed increased ORs but did not reach statistical significance. Planning, administrative, and research work in technical fields showed an inverse association with ALS (OR 0.69, 95% CI 0.48–0.98).

**Table 2:** Results of conditional logistic regression analyses (odds ratios and 95% confidence intervals).

	**Cases *n***	**Controls *n***	**OR**	**95% CI**		***p*** **-Value**	**FDR**
				**Lower**	**Upper**		
Clerical work and other clerical work	313	1,949	ref.				
Building caretaking and cleaning	276	1,483	1.16	0.97	1.39	0.10	ns
Woodwork	174	871	1.22	0.99	1.51	0.06	ns
Forestry work	60	289	1.26	0.93	1.72	0.14	ns
Dock and warehouse work	59	279	1.30	0.96	1.78	0.10	ns
Mining and quarrying work	7	25	1.72	0.73	4.00	0.21	ns
Planning, administrative and research work in the technical fields	40	349	**0.69**	**0.48**	**0.98**	0.04	ns
Packing and wrapping work	45	183	**1.53**	**1.08**	**2.17**	0.02	ns
Laundering, dry cleaning and pressing work	19	65	**1.83**	**1.08**	**3.08**	0.02	ns
Travel service work	7	5	**8.75**	**2.76**	**27.74**	0.001	s

Only selected results showed. The table including the results of all occupational groups is provided as supplementary material.

Benjamini–Hockberg false discovery rate (FDR) ns = non-significant, s = significant. Bold values indicate statistical significance.

In the analyses stratified by sex (data not shown), a statistically significantly increased risk was found among men for postal and telecommunications work (OR 2.34, 95% CI 1.03–5.35) and building caretaking and cleaning work (OR 1.55, 95% CI 1.01–2.40). Increased ORs were also observed among dock and warehouse work (OR 1.56, 95% CI 0.99–2.44), textile work (OR 2.17, 95% CI 0.95–4.94), other professional health care and medical work (OR 2.08, 95% CI 0.92–4.68), and laundering, dry cleaning, and pressing work (OR 6.58, 95% CI 0.90–47.87). Statistically significantly increased risks were found among women for stationary engine and machine work (OR 2.21, 95% CI 1.10–4.45), woodwork (OR 1.57, 95% CI 1.01–2.44), packing and wrapping work (OR 1.47, 95% CI 1.00–2.17), and travel service work (OR 37.7, 95% CI 4.52–314.49). Increased ORs were seen in laundering, dry cleaning, and pressing work (OR 1.67, 95% CI 0.96–2.89).

Stratified analyses (data not shown) by birth cohort (those born in 1915–1934 and those born in 1935–1963) showed that in the younger cohort, woodwork (OR 1.40, 95% CI 1.03–1.89), dock and warehouse work (OR 1.77, 95% CI 1.18–2.66), and travel service work (OR 8.41, 95% CI 2.24–31.65) were associated with an increased risk of ALS. An increased, but not statistically significant risk was found in textile work (OR 1.72, 95% CI 0.97–3.05), in other manufacturing work (OR 1.51, 95% CI 0.99–2.30), in packing and wrapping work (OR 1.56, 95% CI 0.92–2.34), in building caretaking and cleaning work (OR 1.26, 95% CI 0.98–1.63), and in laundering, dry cleaning, and pressing work (OR 2.36, 95% CI 0.98–5.69). A decreased risk was found in the planning, administrative, and research work in technical fields (OR 0.62, 95% CI 0.40–0.99). In the older stratum, a decreased OR was found among transport service work (e.g., road and tram service personnel; OR 0.20, 95% CI 0.05–0.85), while an increased ORs were seen in mining and quarrying work (OR 2.87, 95% CI 1.06–7.80) and travel service work (OR 11.78, 95% CI 1.06–130.98).

Sensitivity analyses were performed by calculating the total number of cases/controls ever/never employed in each occupation and running analyses with these variables (including all occupations until the index date). The results of these calculations support our main findings.

Of the statistically significant associations identified, only travel service work was significant after FDR multiple testing correction.

## Discussion

This study aimed to find out whether there are occupational groups in which the risk of ALS is increased among the Finnish work force. The results showed that the risk was increased in packing and wrapping work, laundering, dry cleaning and pressing work, as well as travel service work. In addition, in sex-stratified analyses, postal and telecommunications work and building caretaking and cleaning work were associated with an increased risk among men, and stationary engine and machine work and woodwork among women (in addition to packing and wrapping work and travel service work).

The increased risk of ALS related to laundering, dry cleaning, and pressing work has not been reported before. However, in a study relying on self-reported exposures, dry cleaning agents have come up as a potential risk factor for ALS (12). A similar finding was made in a study on neurodegenerative diseases including ALS (26). Perchloroethylene is one of the most common solvents used in dry cleaning. It is known to have neurotoxic effects (27) and has recently been found to be associated with ALS among men in a Danish study (28).

Associations of packing and wrapping work with neurodegenerative diseases including motor neuron diseases have been suggested before (29), but we are not aware of studies reporting associations with ALS in particular. Potential exposures in packing and wrapping work may be linked to packaged items/stuff or packaging materials, such as plastics. A plentitude of chemicals is associated with plastic packaging. Typical exposures include solvents, monomers, intermediates, surfactants, plasticizers, stabilizers, biocides, flame retardants, accelerators, and colorants (30). Of these, for example, styrene and flame retardants have been suggested to be associated with ALS (31, 32).

Travel service work was associated with the highest risk of ALS in our study but was based on a small number of cases. Travel service work includes occupations such as pursers and hostesses, who could work, e.g., in an airplane. Recently, Pinkerton et al. (33) reported a doubled risk of ALS among flight attendants. Exposures that have been studied in relation to cabin work include, e.g., radiation, ionization, low-frequency magnetic fields, and tricresyl phosphate (18, 34). Of these, low-frequency magnetic fields have been suggested as a risk factor for ALS and e.g., tricresyl phosphate is a known neurotoxicant (33). Thus, this association also warrants further study focusing on the potential specific exposures.

In our study, the results were partly different for women and men. This may be explained by the fact that we studied 70 occupational groups, all of which included several occupations. Men and women in these groups could work in different occupations. In some occupations, such as mining and quarrying work, the sex distribution was particularly unequal (no women), limiting our ability to consider sex differences.

We also found some potential birth cohort effects in our exploratory stratified analyses. For example, mining and quarrying work showed an elevated risk only among those born before 1935. This suggests that the risk occupations and related exposures may change over time or that the latency time is long.

The association of ALS with dock and warehouse work, which was found among men and among the younger birth cohort, was new. Diesel exhaust exposure has been found to be associated with ALS and may provide a link between dock and warehouse work and ALS *via* several possible pathomechanisms, as suggested by Dickerson et al. (20). Diesel exhaust may also be the link between stationary engine and machine work and ALS among women and mining and quarrying work and ALS among men. However, mining and quarrying work may include several other potential exposures as well, such as crystalline silica dust, which has also shown associations with ALS (21). Overall, the exposures in the occupations with increased risk of ALS and potential pathomechanisms warrant further studies.

The occupational risk groups found in our study were mostly different compared to both the Danish and Swedish register-based case–control studies (10, 11). Agriculture, hunting, forestry, and fishing, which were found to be associated with ALS among men in the Danish study, were not associated with ALS in our study. However, in the Danish study, these occupations were associated with ALS only when combined as one group. Similar to our study, they did not show statistically significant associations with ALS separately. In our material construction work, precision-tool manufacturing or glass, tile, and pottery work, of which the two latter were found to be associated with ALS in the Swedish study, were not associated with ALS. However, forestry work showed an increased risk in our study, even though it did not reach statistical significance and if mining and quarrying work is considered as part of construction work, then our results partly support the previous findings of the Danish study.

Finland, Denmark, and Sweden are all Nordic countries with geographic vicinity and likely to have largely similar occupational distributions and exposures, but the results obtained in these countries vary country by country. This may be due to several differences in the selection of study samples, study methods, and occupational classification. The Danish sample was smaller and included those who were born in 1939 or later. Thus, the sample was clearly younger than ours or the Swedish sample, which means that the active time in work–life is in a later period than in our study or the Swedish study. The occupational distribution as well as work exposures may change over time, an observation that is supported by our birth cohort analyses. In addition, a large part of those who fall ill with ALS in an older age is outside the scope of the Danish study, which may additionally affect the results, because it may e.g., select out occupational risks associated with longer latency times. In addition, the Danish study utilized the whole occupational history, which resulted in a different statistical strategy. They compared ever exposed to those never exposed to the particular occupation. Our reference group was all the time the same, ie. “clerical work and other office work” and we chose to select the longest-held occupation as the subject to review. The Swedish study design resembled more closely ours and had the sample and occupational information largely from the same period and they too had a stable reference group in the analyses: “Administrative and clerical work.” However, the occupational classification in the Swedish study seems to be different (more robust) from ours. That study also took several occupations per person into account while we focused on the longest-held occupation.

### Strengths and weaknesses of the study

This was a large-scale register-based study including all ALS cases from death records in Finland during the selected study period and their controls matched for birth year and sex. Information on occupation was obtained from the registers of Statistics Finland. The strength of the register study is that it is not prone to recall bias and enables a large sample size, which is needed to study a large variety of occupations. However, even though we had all ALS cases that were reported in Finland during the study period, the confidence intervals were wide in some occupational groups due to a small number of cases in these groups. Thus, the results should be seen as suggestive.

The weaknesses of the data related to the precision of occupational information. Part of the study subjects had several occupations during the study period. We used the most frequent occupation from the subject's work history in the analyses. However, it is not yet known how long an exposure period is needed to increase the risk of ALS, or to what extent such exposure times might vary by occupation. Thus, it is possible that some other occupation in the history of the subjects is related to ALS rather than the most frequent occupation. Yet, the sensitivity analyses, which were done with the variables including the total number of cases/controls ever/never employed in each occupation, support our main findings. Still, a study considering the whole occupational history and being able to calculate actual exposure times per occupation would be needed to shed more light on this.

We had participants born over a period of 39 years and the amount of occupational information varied per person. It is possible that we did not catch the most frequent occupation in the complete occupational history for all study subjects.

We studied the association of ALS with occupations with registered information. We acknowledge that there are plenty of non-occupational factors that may contribute to the onset of ALS, such as health behavior or those physical and chemical exposures that are not related to occupation. In addition, genetic analyses were outside the scope of this study.

## Conclusion

This study pinpoints occupations related to ALS. Future research should deepen our understanding of the role of these occupations as risk factors for ALS, focus on the cumulative effects of physical and chemical exposures to these occupations, and learn about meaningful exposure times. In addition, as ALS is assumed to be a multifactorial disease, a simultaneous study of occupational exposures and other potential risk factors, such as other medical conditions would be preferable.

This study covers the occupations typical to 1975–1993. Since then, the major changes in occupational distribution have occurred, as societies are moving toward an increased amount of information work, and the exposures related to occupations change simultaneously. The potential effects of the changing work–life and exposure to deaths due to ALS will only be seen in the future.

## Data availability statement

The datasets presented in this article are not readily available because national data protection legislation prevents the sharing of these datasets with third parties. Requests to access the datasets should be directed to PS, peppiina.saastamoinen@helsinki.fi.

## Ethics statement

This study was reviewed and approved by the Ethics Committee of the Department of Public Health, University of Helsinki. Written informed consent for participation was not required for this study in accordance with the national legislation and the institutional requirements.

## Author contributions

PS initiated the study, made plans, was responsible for the data management, made analyses, and drafted first manuscript. HL took part on the planning of the study, provided neurological expertise on ALS, reviewed death certificates and listed all non-ALS cases that needed to be removed from the data, and critically reviewed the manuscript. PL-A took part on the planning of the study, provided expertise on occupational epidemiology, and critically reviewed the manuscript. OR provided expertise on epidemiology and critically reviewed the manuscript. All authors contributed to the article and approved the submitted version.

## Funding

PS was supported by the Finnish Work Environment Fund (grant #116254). Lihastautiliitto supported data collection.

## Conflict of interest

The authors declare that the research was conducted in the absence of any commercial or financial relationships that could be construed as a potential conflict of interest.

## Publisher's note

All claims expressed in this article are solely those of the authors and do not necessarily represent those of their affiliated organizations, or those of the publisher, the editors and the reviewers. Any product that may be evaluated in this article, or claim that may be made by its manufacturer, is not guaranteed or endorsed by the publisher.

## References

[1] Longinetti E Fang F. Epidemiology of amyotrophic lateral sclerosis: an update of recent literature. Curr Opin Neurol. (2019) 32:771–6. 10.1097/WCO.0000000000000730 31361627 PMC6735526

[2] Logroscino G Traynor BJ Hardiman O Chiò A Mitchell D Swingler RJ. Incidence of amyotrophic lateral sclerosis in Europe. J Neurol Neurosurg Psychiatry. (2010) 81:385–90. 10.1136/jnnp.2009.183525 19710046 PMC2850819

[3] del Aguila MA Longstreth WT Jr McGuire V Koepsell TD van Belle G. Prognosis in amyotrophic lateral sclerosis: a population-based study. Neurology. (2003) 60:813–9. 10.1212/01.WNL.0000049472.47709.3B 12629239

[4] Chiò A Logroscino G Hardiman O Swingler R Mitchell D Beghi E. Prognostic factors in ALS: a critical review. Amyotroph Lateral Scler. (2009) 10:310–23. 10.3109/17482960802566824 19922118 PMC3515205

[5] Riancho J Bosque-Varela P Perez-Pereda S Povedano M de Munaín AL Santurtun A. The increasing importance of environmental conditions in amyotrophic lateral sclerosis. Int J Biometeorol. (2018) 62:1361–74. 10.1007/s00484-018-1550-2 29713861

[6] Zufiría M Gil-Bea FJ Fernandez-Torrón R Poza JJ Muñoz-Blanco JL Rojas-García R. ALS: A bucket of genes, environment, metabolism and unknown ingredients. Prob Neurobiol. (2016) 142:104–29. 10.1016/j.pneurobio.2016.05.004 27236050

[7] Al-Chalabi A Hardiman O. The epidemiology of ALS: a conspiracy of genes, environment and time. Nat Rev Neurol. (2013) 9:617–28. 10.1038/nrneurol.2013.203 24126629

[8] Vasta R Chia R Traynor B Chiò A. Unravelling the complex interplay between genes, environment, and climate in ALS. eBioMedicine. (2022) 75:103795. 10.1016/j.ebiom.2021.103795 34974309 PMC8728044

[9] Goutman SA Hardiman O Al-Chalabi A Chió A Savelieff MG Kiernan MC. Emerging insights into the complex genetics and pathophysiology of amyotrophic lateral sclerosis. Lancet Neurol. (2022) 21:465–679. 10.1016/S1474-4422(21)00414-2 35334234 PMC9513754

[10] Dickerson AS Hansen J Kioumourtzoglou MA Specht AJ Gredal O Weisskopf MG. Study of occupation and amyotrophic lateral sclerosis in a Danish cohort. Occup Environ Med. (2018) 75:630–8. 10.1136/oemed-2018-105110 29941657 PMC6445390

[11] Peters TL Kamel F Lundholm C Feychting M Weibull CE Sandler DP. Occupational exposures and the risk of amyotrophic lateral sclerosis. Occup Environ Med. (2017) 74:87–92. 10.1136/oemed-2016-103700 27418175 PMC5451107

[12] Fang F Quilan P Ye W Barber MK Umbach DM Sandler DP. Workplace exposures and the risk of amyotrophic lateral sclerosis. Environ Health Perspect. (2009) 177:1387–92. 10.1289/ehp.0900580 19750102 PMC2737014

[13] D'Ovidio F. d‘Errico A Calvo A Costa G Chió A. Occupations and amyotrophic lateral sclerosis: are jobs exposed to the general public at higher risk? Eur J Public Health. (2017) 27:643–7. 10.1093/eurpub/ckx006 28201634

[14] Zhou H Chen G Chen C Yu Y Xu Z. Associations between extremely low-frequency electromagnetic fields occupations and amyotrophic lateral sclerosis: a meta-analysis. PLoS ONE. (2012) 7:e48354. 10.1371/journal.pone.0048354 23189129 PMC3506624

[15] Yu Y Su F-C Callaghan BC Goutman SA Batterman SA Feldman EL. Environmental risk factors and amyotrophic lateral sclerosis (ALS): a case-control study of ALS in Michigan. PLoS ONE. (2014) 9:e101186. 10.1371/journal.pone.0101186 24979055 PMC4076303

[16] Beard JD Kamel F. Military service, deployments, and exposures in relation to amyotrophic lateral sclerosis etiology and survival. Epidemiol Rev. (2015) 37:55–70. 10.1093/epirev/mxu001 25365170 PMC4325667

[17] Fisher H Kheifets L Huss A Peters TL Vermeulen R Ye W. Occupational exposure to electric shocks and magnetic fields and amyotrophic lateral sclerosis in Sweden. Epidemiology. (2015) 26:824–30. 10.1097/EDE.0000000000000365 26414853

[18] Koeman T Slottje P Schouten L Peters S Huss A Veldink J. Occupational exposure and amyotrophic lateral sclerosis in a prospective cohort. Occup Environ Med. (2017) 74:578–85. 10.1136/oemed-2016-103780 28356332

[19] Seals RM Kioumourtzoglou MA Gredal O Hansen J Weisskoft MG. Occupational formaldehyde and amyotrophic lateral sclerosis. Eur J Epidemiol. (2017) 32:893–9. 10.1007/s10654-017-0249-8 28585120 PMC5681368

[20] Dickerson AS Hansen J Gredal O Weisskopf MG. Amyotrophic lateral sclerosis and exposure to diesel exhaust in a Danish cohort. Am J Epidemiol. (2018) 187:1613–22. 10.1093/aje/kwy069 29590300 PMC6070035

[21] Visser AE D'Ovidio F Peters S Vermeulen RC Beghi E Chió A. Multicentre, population-based, case-control study of particulates, combustion products and amyotrophic lateral sclerosis risk. J Neurol Neurosurg Psychiatry. (2019) 90:854–60. 10.1136/jnnp-2018-319779 30850472

[22] Meng E Mao Y Yao Q Han X Li X Zhang K. Population-based study of environmental/occupational lead exposure and amyotrophic lateral sclerosis: a systematic review and meta-analysis. Neurol Sci. (2020) 41:35–40. 10.1007/s10072-019-04067-z 31578652

[23] Dickerson AS Hansen J Specht AJ Gredal O Weisskopf MG. Population-based study of amyotrophic lateral sclerosis and occupational lead exposure in Denmark. Occup Environ Med. (2019) 76:208–14. 10.1136/oemed-2018-105469 30705111 PMC6433465

[24] Gunnarsson LG Bodin L. Amyotrophic lateral sclerosis and occupational exposures: a systematic literature review and meta-analyses. Int J Environ Res Public Health. (2018) 15:2371. 10.3390/ijerph15112371 30373166 PMC6265680

[25] Wang MD Little J Gomes J Cashman NR Krewski D. Identification of risk factors associated with onset and progression of amyotrophic lateral sclerosis using systematic review and meta-analysis. Neurotoxicology. (2017) 61:101–30. 10.1016/j.neuro.2016.06.015 27377857

[26] Cannon J Greenamyre T. The role of environmental exposures in neurodegeneration and neurodegenerative diseases. Toxicol Sci. (2011) 124:225–50. 10.1093/toxsci/kfr239 21914720 PMC3216414

[27] Ceballos DM Fellows KM Evans AE Janulewicz PA Lee EG Whittaker SG. Perchloroethylene and dry cleaning: It's time to move the industry to safer alternatives. Front Public Health. (2021) 9:638082. 10.3389/fpubh.2021.638082 33748070 PMC7973082

[28] Dickerson AS Hansen J Thompson S Gredal O Weisskopf MG. A mixtures approach to solvent exposures and amyotrophic lateral sclerosis: a population-based study in Denmark. Eur J Epidemiol. (2020) 35:241–9. 10.1007/s10654-020-00624-5 32193761 PMC7176306

[29] Schulte PA Burnett CA Boeniger MF Johnson J. Neuro-degenerative diseases: occupational occurrence and potential risk factors, 1982 to 1991. Am J Public Health. (1996) 86:1281–8. 10.2105/AJPH.86.9.1281 8806381 PMC1380592

[30] Groh KJ Backhaus T Carney-Almroth B Geueke B Inostroza PA Lennquist A. Overview of known plastic packaging-associated chemicals and their hazards. Sci Total Environ. (2019) 651:3253–68. 10.1016/j.scitotenv.2018.10.015 30463173

[31] Malek AM Barchowsky A Bowser R Heiman-Patterson T Lacomis D Rana S. Exposure to hazardous air pollutants and the risk of amyotrophic lateral sclerosis. Environ Pollut. (2015) 197:181–6. 10.1016/j.envpol.2014.12.010 25544309

[32] Su F-C Goutman SA Chernyak S Mukherjee B Callaghan BC Batterman S. Associations of environmental toxins with amyotrophic lateral sclerosis. JAMA Neurol. (2016) 73:803–11. 10.1001/jamaneurol.2016.0594 27159543 PMC5032145

[33] Pinkerton L Hein M Grajewski B Kamel F. Mortality from neurodegenerative diseases in a cohort of US flight attendants. Am J Ind Med. (2016) 59:532–7. 10.1002/ajim.22608 27184412 PMC4915549

[34] de Boer J Antelo A van der Veen I Brandsma S Lammmertse N. Tricresyl phosphate and the aerotoxic syndrome of flight crew members – current gaps in knowledge. Chemosphere. (2015) 119 (suppl):S58–61. 10.1016/j.chemosphere.2014.05.015 24925093

